# Restricted intra-embryonic origin of bona fide hematopoietic stem cells in the chicken

**DOI:** 10.1242/dev.151613

**Published:** 2017-07-01

**Authors:** Laurent Yvernogeau, Catherine Robin

**Affiliations:** 1 Hubrecht Institute-KNAW and University Medical Center Utrecht, Uppsalalaan 8, Utrecht 3584 CT, The Netherlands; 2 Department of Cell Biology, University Medical Center Utrecht, Utrecht 3584 EA, The Netherlands

**Keywords:** Embryo, Chicken, Aorta, Hematopoietic stem cells, Hematopoietic clusters, Chorio-allantoic membrane transplantation

## Abstract

Hematopoietic stem cells (HSCs), which are responsible for blood cell production, are generated during embryonic development. Human and chicken embryos share features that position the chicken as a reliable and accessible alternative model to study developmental hematopoiesis. However, the existence of HSCs has never been formally proven in chicken embryos. Here, we have established a complete cartography and quantification of hematopoietic cells in the aorta during development. We demonstrate the existence of bona fide HSCs, originating from the chicken embryo aorta (and not the yolk sac, allantois or head), through an *in vivo* transplantation assay. Embryos transplanted *in ovo* with GFP embryonic tissues on the chorio-allantoic membrane provided multilineage reconstitution in adulthood. Historically, most breakthrough discoveries in the field of developmental hematopoiesis were first made in birds and later extended to mammals. Our study sheds new light on the avian model as a valuable system to study HSC production and regulation *in vivo*.

## Abstract (precis)

**Summary:** Bona fide hematopoietic stem cells originate from the aorta – and not the yolk sac, allantois or head – during embryonic development in chick.

## INTRODUCTION

Over the past century, the avian has been a pioneer animal model for major breakthrough discoveries made in the field of developmental hematopoiesis that were later extended to other vertebrates (Jaffredo and Yvernogeau, 2014; Le Douarin and Dieterlen-Lièvre, 2013). The avian embryo is an excellent experimental model owing to its accessibility in the egg throughout development. The flat layout of the embryo on the top of the yolk sac (YS) allowed the creation of chimeras to trace cell fate *in vivo* (Le Douarin, 1969; Le Douarin and Jotereau, 1975; Martin, 1972). Of importance was the sophisticated YS chimera, created by engrafting a whole quail embryo on the chicken YS of a comparable developmental stage (Beaupain et al., 1979; Dieterlen-Lievre, 1975). In the 1970s, Moore and Owen proposed the YS as the exclusive site of hematopoietic stem cell (HSC) production in both avian and mammalian embryos (Moore and Owen, 1967a,b). However, the use of avian YS chimeras provided the first experimental proof that cells found 11 days post-grafting in the spleen and thymus rudiment (granulocytes or erythrocytes, and lymphocytes, respectively) were of quail intra-embryonic origin (Dieterlen-Lievre, 1975). B and T lymphocytes (observed at 18 days post-grafting) and erythrocytes (detected in the blood at 4 weeks post-hatching) were also of embryonic origin in allogenic chimeras (chicken-chicken YS-embryo) (Lassila et al., 1978, 1982). Importantly, the YS either was not contributing or was providing only a transient wave of blood cells. The avian model therefore proved the long-disputed intra-embryonic origin of the adult hematopoietic system and highlighted the region of the dorsal aorta as the prospective hematopoietic stem/progenitor cell source (Cormier and Dieterlen-Lievre, 1988; Dieterlen-Lièvre and Martin, 1981). Noteworthy, donor cell contribution was only determined in the short term (between few days post-grafting to up to 6 weeks post-hatching) (Lassila et al., 1979) or in the long term (up to 20 weeks post-hatching), but solely to lymphocytes, which were tested indirectly via their response to antigens and mitogens (Martin et al., 1979). Thus, it is difficult to ascertain whether HSCs or long-lived committed progenitors engrafted in chimeras. The existence of bona fine HSCs in the chicken embryo is therefore yet to be proven.

An important observation, initially made in the chicken embryo, revealed the presence of hematopoietic cell clusters (thereafter referred to as intra-aortic hematopoietic clusters or IAHCs) intimately attached to the aortic wall (Dantschakoff, 1909; Jordan, 1917). They are a common feature of specific early developmental stages of almost all vertebrate embryos (Dieterlen-Lievre et al., 2006; Garcia-Porrero et al., 1995; Tavian et al., 1996; Walmsley et al., 2002). In mice, IAHCs are present when the first HSCs (identified in transplantation assays) start to be detected in the aorta of the aorta-gonad-mesonephros (AGM) region, the umbilical and vitelline arteries, and the vascular labyrinth of the placenta at embryonic day (E)10.5-E11 of development (de Bruijn et al., 2000; Medvinsky and Dzierzak, 1996; Müller et al., 1994; Ottersbach and Dzierzak, 2005; Rhodes et al., 2008; Yokomizo and Dzierzak, 2010). Based on these observations and on the absence of IAHCs in *Runx1*^−/−^ embryos (also devoid of HSCs but containing primitive erythroid precursors), it is comprehensively accepted that HSCs and progenitors reside in IAHCs (Lacaud et al., 2002; North et al., 2002, 1999; Cai et al., 2000; Okuda et al., 1996; Wang et al., 1996). In chicken embryo, IAHCs derive directly from specialized endothelial cells [hemogenic endothelial (HE) cells] integrated in the endothelial layer of the aortic wall (Jaffredo et al., 2000, 1998). *In vivo* lineage-tracing experiments and live confocal imaging observations confirmed the HE origin of IAHCs and HSCs in zebrafish and mouse embryos, which are generated via the so-called endothelial-to-hematopoietic transition (EHT) (Bertrand et al., 2010; Boisset et al., 2010; Chen et al., 2009; Kissa and Herbomel, 2010; Lam et al., 2010; Zovein et al., 2008). High-resolution 3D microscopic visualization of transparent mouse embryos has provided a precise cartography and quantification of IAHC cells in arteries (Yokomizo and Dzierzak, 2010). Such analysis is missing in other vertebrate species. In mouse, IAHCs start to appear in the aorta at E9.5, peak in number (≈700 cells per aorta) at E10.5 and then decrease until E14.5. Transplantations performed with limiting cell dilutions led to estimates of fewer than three HSCs per mouse or human AGM (Ivanovs et al., 2011; Kumaravelu et al., 2002). Most IAHC cells are in fact HSC precursors (pre-HSCs), able to mature into functional HSCs when transplanted in permissive recipients (e.g. newborn, immunodeficient adult mice) or after a step of culture with OP9 cells (in AGM reaggregates) (Boisset et al., 2015; Rybtsov et al., 2016, 2011; Taoudi et al., 2008). *In vivo*, the HSC pool is constituted by HSC proliferation and/or IAHC pre-HSC maturation after migration to the placenta and/or fetal liver at mid-gestation (Gekas et al., 2005; Kumaravelu et al., 2002; Ottersbach and Dzierzak, 2005; Rybtsov et al., 2016), before colonizing the bone marrow (BM) prior to birth (Christensen et al., 2004). In birds, IAHC cells ingress underneath the aorta to form para-aortic foci (PAFs) (Dieterlen-Lièvre and Martin, 1981; Jaffredo et al., 2000), before colonizing the definitive hematopoietic organs [thymus, bursa of Fabricius (bird B cell organ), spleen and BM] (Dunon et al., 1998, 1999; Lassila et al., 1980).

In mammals, the HSC activity is tested upon transplantation into irradiated adult recipients. A major drawback of the avian model is the lack of similar assays where both the long-term and multilineage potential of dissected tissues or cells can be tested. The irradiation of adult chicken is challenging and the necessity to inject over ten million cells into the host recipient to evaluate their functionality is unrealistic with embryonic cells (Lassila et al., 1979; Samarut and Nigon, 1975). Although YS chimeras are only designed to graft whole embryos, a technique for transplantation of embryonic organs (e.g. thymus, spleen) onto the chorio-allantoic membrane (CAM) was described in the 1970s to study the hematopoietic potential of organs (Metcalf and Moore, 1971). CAM transplantations of forelimbs recently demonstrated the timing of forelimb colonization by presomitic-derived angioblasts and myoblasts (Yvernogeau et al., 2012). CAM transplantations have two main advantages. The dissected organ/tissue to test can no longer be colonized by cells from the donor chicken embryo after CAM transplantation. Moreover, the grafted tissue can grow after connection to the vascular system of the recipient (accessible in the CAM). Importantly, GFP transgenic chickens are now available for an easy detection and tracking of donor cells (McGrew et al., 2004).

In the present study, we provide the first cartography and quantification of IAHCs in the aorta of chicken embryos at different time points of development. By performing whole-mount fluorescent immunostaining and 3D reconstruction of whole aorta and chicken embryos, we show that IAHC emergence is a regulated process, both in time and space. IAHC emergence starts at E2.25 along the anterior to posterior axis of the embryo as development unfolds. IAHCs remain restricted to the ventral floor of the anterior portion of the aorta (from the aortic arches to the aorta-vitelline arteries connection). PAF cell emergence follows IAHC emergence. Because very few PAF cells proliferate, it seems that PAFs are not a proliferation site but rather a maturation site. Using CAM transplantation assays and GFP transgenic chicken embryos, we have proved the existence of HSCs in the chicken embryo. HSCs originate from the AGM region and not from the YS, allantois or head. Altogether, we provide a precise and complete spatial and temporal cartography of IAHCs and PAFs along the aorta during embryonic development and demonstrate that, similar to mammals, HSCs originate in the AGM region of the chicken embryo.

## RESULTS

### Mapping and quantification of IAHC and PAF cells in the dorsal aorta during chicken embryonic development

**Fig. 1.**
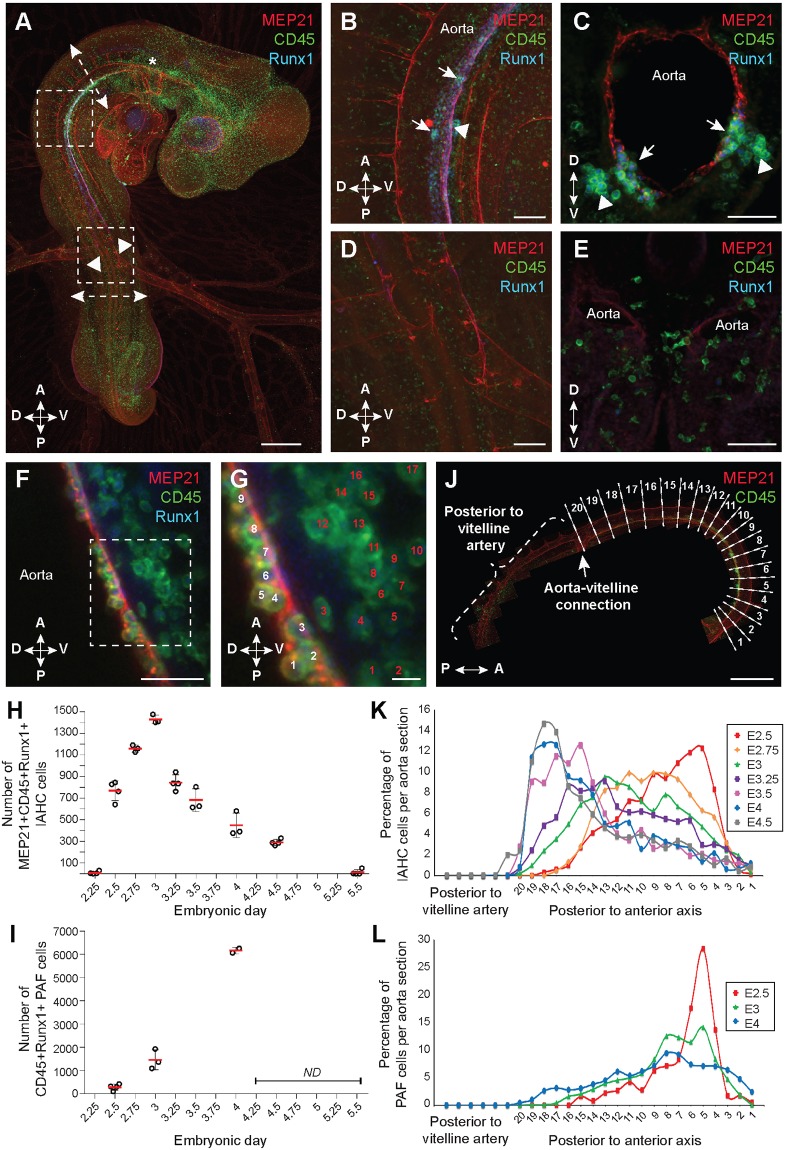
**Spatial and temporal emergence of IAHC and PAF cells in the chicken embryonic aorta.** (A) Whole-mount of E3 chicken embryo stained using anti-MEP21 (red), anti-Runx1 (blue) and anti-CD45 (green) antibodies. Asterisk, aortic arches; arrowheads, aorta-vitelline connection; upper boxed area is enlarged in B; lower boxed area is enlarged in D; upper and lower dashed arrows indicate the locations of the cross-sections shown in C and E, respectively. (B,C) Enlarged views of the anterior part of the aorta. Sagittal (B) and transverse (C) views. (D,E) Enlarged views of the posterior part of the aorta. Sagittal (D) and transverse (E) views. The aorta is paired in the posterior region of the E3 embryo. Arrowheads, PAF cells; arrows, IAHC cells. (F) Enlarged view of the anterior part of an E3 chicken aorta stained using anti-MEP21 (red), anti-Runx1 (blue) and anti-CD45 (green) antibodies. (G) Enlarged view of the anterior part of the aorta boxed in F, illustrating the counting of MEP21^+^Runx1^+^CD45^+^ IAHC (white numbers) and Runx1^+^CD45^+^ PAF (red numbers) cells. Images in A,B,D are related to Movie 1. (H) The total number of MEP21^+^Runx1^+^CD45^+^ IAHC cells per aorta between embryonic day (E)2.25 and E5.5. (I) The total number of Runx1^+^CD45^+^ PAF cells per aorta at E2.5, E3 and E4. (J) Maximal projection of the aorta stained using anti-MEP21 (red) and anti-CD45 (green) antibodies. The inter-somitic vessel (ISV) position is used to virtually subdivide the aorta into sections (along the anterior to posterior axis, 1 to 20 sections). (K) Percentage of MEP21^+^Runx1^+^CD45^+^ IAHC cells per aorta section between E2.5 and E4.5. (L) Percentage of Runx1^+^CD45^+^ PAF cells per aorta section at E2.5, E3 and E4. Scale bars: 500 µm in A,J; 100 µm in B,D; 50 µm in C,E,F; 10 µm in G. A, anterior; P, posterior; D, dorsal; V, ventral.

To establish the complete cartography and quantification of IAHC cells along the aorta at different time points of development, whole-mount fluorescent immunostaining was performed on chicken embryos. With this approach, cell organization is visible along the entire aorta without disrupting the integrity of the tissue or dislodging cells, as might occur during embryo sectioning. Chicken embryos were co-stained with antibodies against MEP21 (endothelial marker), Runx1 (HE and hematopoietic stem/progenitor cell marker), and CD45 (hematopoietic cell marker). After confocal imaging and 3D reconstruction of whole embryos, the precise location of MEP21^+^Runx1^+^CD45^+^ IAHC cells was nicely visible along the dorsal aorta (Fig. 1A; Movie 1; E3 embryo shown as an example). IAHC cells were restricted to the most anterior part of the aorta, delimited by the aortic arches (Fig. 1A, asterisk) and the connection between the aorta and the vitelline arteries (Fig. 1A, arrowheads). In this region, IAHC cells were exclusively present in the ventral side of the aorta (Fig. 1A-C; arrows). In contrast, no IAHC cells were visible in the posterior part of the aorta (after the aorta-vitelline connection, Fig. 1D,E), whereas some MEP21^+^Runx1^+^CD45^−^ HE cells were present (Movie 1). Noteworthy, the two bilateral aortic anlagen were not fused yet in the posterior region of E3 embryos. MEP21^−^Runx1^−^CD45^+^ cells visible outside the aorta were mature hematopoietic cells.

To estimate the total number of IAHC cells in the aorta, chicken embryos were isolated from E2 to E5.5 (three or four embryos per time point), stained and imaged as described above. All MEP21^+^Runx1^+^CD45^+^ IAHC cells were counted (Fig. 1F,G; white numbers). Interestingly, even though no IAHCs were present at E2 (time point close to the establishment of blood circulation), HE Runx1^+^ cells started to be detected lining both sides of the paired aortas (Movie 2). IAHC cells were detected at E2.25, when the paired aortas started to fuse (9±14 IAHC cells/aorta) (Fig. 1H; Movie 3). The number of IAHC cells quickly increased to reach a peak at E3 (1429±40 IAHC cells/aorta). The number of cells then gradually decreased with only very few IAHC cells detectable at E5.5 (13±25 IAHC cells/aorta) (Fig. 1H). We also estimated the total number of PAF cells in E2.5, E3 and E4 embryos (Fig. 1F,G; red numbers). After E4, PAF cells were too numerous to be counted accurately. Runx1^+^CD45^+^ PAF cells were located in the mesenchyme underneath the aorta, as shown as an example in an E3 embryo (Fig. 1A-C, arrowheads; Movie 1). PAF cells were detected from E2.5 onwards (282±132 PAF cells/aorta) (Fig. 1I). Their number then increased continuously until E4 (6160±143 PAF cells/aorta). Altogether, our data provide a precise mapping and quantification of IAHC and PAF cells in the aorta of the chicken embryo during development.

### IAHC and PAF cells emerge as a wave from the anterior to posterior axis of the aorta with low proliferation

The total number of IAHC cells does not provide spatial information on the distribution of these cells along the aorta and whether this distribution changes during embryonic development. To answer these questions, the aorta of the previously used embryos was virtually subdivided into small sections delimited by the inter-somitic vessels (ISV) (an E3 embryo is shown as an example in Fig. 1J; sections 1 to 20). The region of the aorta, which was posterior to the aorta-vitelline connection, was not subdivided and was considered as a whole (as no IAHC or PAF cells were detected there; Fig. 1A,D,E; Movie 1). IAHC cells were counted in each successive virtual section of the aorta (between E2.5 and E4.5). Knowing the total number of IAHC cells per aorta for each embryo, we then calculated the percentage of IAHC cells per section for all developmental time points (percentage=IAHC cell number per section/IAHC cell number per aorta×100) (Fig. 1K). IAHC cells were located in the most anterior region of the aorta at E2.5, where the percentages of IAHC cells peak in ISV sections 5 and 6. As the embryonic development unfolds, IAHC cell distribution shifted along the anterior to posterior axis, with IAHC cells locating closer to the aorta-vitelline connection (Fig. 1K). By E4.5, IAHC cells were mostly located just before (anterior to) the aorta-vitelline connection between sections 16 and 19. Noteworthy, few IAHC cells (2%) were observed just after (posterior to) the aorta-vitelline connection exclusively at E4.5 (Fig. 1K). We never observed IAHCs in the vitelline arteries (Movies 1, 3 and 5, see the close-ups of the aorta-vitelline connection) or in the allantois (Movie 5). Similar to IAHC cells, the distribution of Runx1^+^CD45^+^ PAF cells in the aorta was also determined in embryos isolated at E2.5, E3 and E4 (Fig. 1L). At E2.5, the highest percentages of PAF cells were observed in the most anterior region of the aorta. PAF cells were more homogeneously distributed along the aorta (before the aorta-vitelline connection) at E3 and E4 (Fig. 1L; Movie 4). Overall, our data show that IAHC distribution in the aorta during embryonic development is an active and regulated process that leads to a progressive and controlled shift of IAHC cell emergence from the anterior to the posterior part of the embryo. PAF cells also initially appeared in the anterior part of the aorta with a more homogeneous distribution as the embryo grows.

To evaluate whether IAHC cell production results of cell proliferation, whole chicken embryos were stained using anti-MEP21, anti-Runx1, anti-CD45 and anti-PHH3 (mitosis-specific marker phospho-histone H3) antibodies (Fig. S1A; Movie 6). Embryos from three specific embryonic developmental stages were analyzed: E2.5 (when the number of IAHC cells is expanding), E3 (at the peak of IAHC cell number) and E4 (when the number of IAHC cells is decreasing) (three or four embryos per time point). Confocal imaging of four-color whole-mount immunostained embryos and 3D reconstruction allowed us to count the total number of IAHC cells (MEP21^+^Runx1^+^CD45^+^) and proliferating IAHC cells (MEP21^+^Runx1^+^CD45^+^PHH3^+^) per aorta (Fig. S1A-G). This ratio was used to calculate the mitotic index at each developmental stage (Fig. S1N). Very few proliferating IAHC cells were observed at all developmental time points, as shown by the mitotic indices (Fig. S1N; 5.4±1.3% at E2.5, 5.5±1.2% at E3, and 1±0.6% at E4). The numbers of proliferating IAHC cells and total IAHC cells were then counted per aorta section to examine the distribution of proliferating IAHC cells along the aorta as described above (Fig. S1O). Proliferating IAHC cells were distributed along the anterior to posterior axis as the embryo developed (Fig. S1O) following the IAHC cell distribution (Fig. 1K). Similar to IAHC cells, the mitotic index of PAF cells was calculated at E2.5, E3 and E4 (Fig. S1N; Movie 6) after counting the total number of PAF cells (Runx1^+^CD45^+^) and proliferating PAF cells (Runx1^+^CD45^+^PHH3^+^) per aorta (Fig. S1H-M). Despite a massive increase in PAF cells over time (Fig. 1I), the mitotic index was low at all developmental time points (Fig. S1N; 2.8±1.4% at E2.5, 4.1±0.4% at E3 and 2.9±0.3% at E4). The precise PAF cell counting in successive individual aorta sections revealed that proliferating PAF cells were more abundant in the anterior part of the aorta at E2.5, E3 and E4 (Fig. S1P), following the PAF cell distribution (Fig. 1L; Movie 4). Overall, our data show that IAHC and PAF cell emergence is a dynamic and organized process, occurring with low proliferation. Whereas the IAHC cell production shifts along the anterior to posterior side of the developing embryo, PAF cells remain mainly in the anterior part of the embryo (Movie 4).

### CAM transplantation allows the independent growth of dissected embryonic hematopoietic tissues *in ovo*

**Fig. 2.**
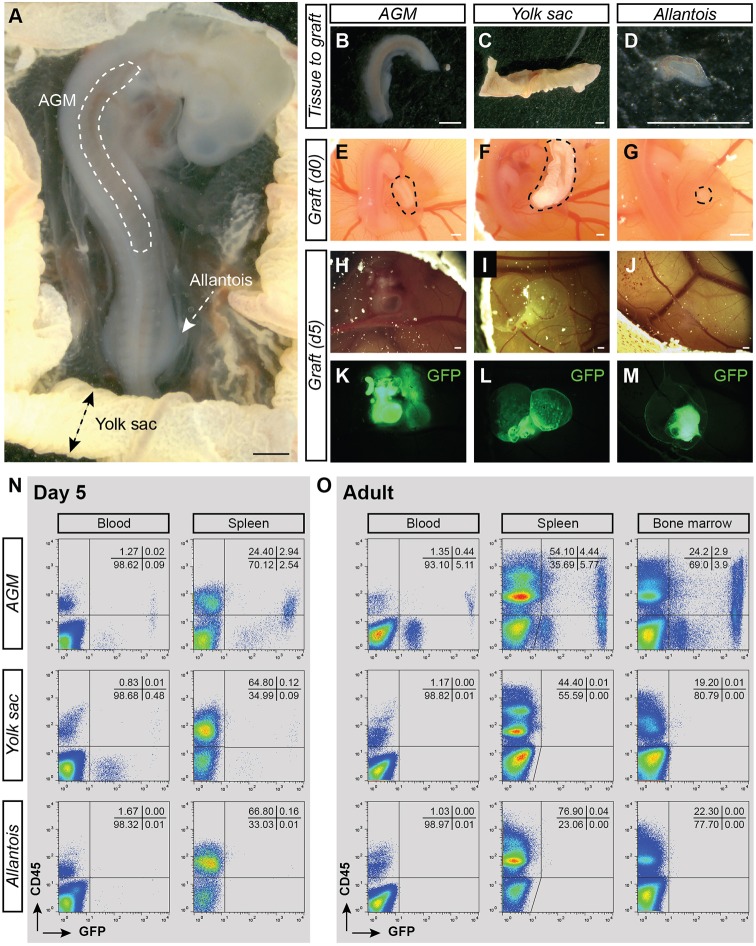
**CAM transplantations allow the autonomous development of dissected AGM, yolk sac and allantois from E3 chicken embryos.** (A) Transmitted light picture of E3 GFP^+^ chicken embryo. The locations of the AGM region, yolk sac (YS) and allantois are indicated. (B-D) Transmitted light pictures of the AGM (B), YS (C) and allantois (D) after dissection. (E-G) Transmitted light pictures of the AGM (E), YS (F) and allantois (G) shown in B-D after transplantation in the CAM of an E4 recipient embryos (*in ovo* views). Dashed areas indicate the position of grafted tissues. (H-J) Transmitted light pictures of the AGM (H), YS (I) and allantois (J) CAM at 5 days post-transplantation. (K-M) Fluorescent pictures of the AGM (K), YS (L) and allantois (M) CAM shown in H-J. GFP, green. (N) Flow cytometry analysis showing donor-cell contribution (GFP) in blood and spleen of AGM (top plots), YS (middle) and allantois (bottom) CAM recipients at 5 days post-transplantation. Cells were stained with anti-CD45 antibody (donor hematopoietic cells: GFP^+^CD45^+^). Percentages of each viable population are indicated per quadrant. (O) Flow cytometry analysis showing donor-cell contribution (GFP) in blood, spleen and BM of AGM (top plots), YS (middle) and allantois (bottom) CAM recipients at 5 months post-transplantation. Cells were stained with anti-CD45 antibody (donor hematopoietic cells: GFP^+^CD45^+^). The analyzed tissue CAM recipients are shown in Table 2. Scale bars: 500 µm.

To evaluate the presence of HSCs in the chicken embryo, chorio-allantoic membrane (CAM) transplantations were performed. This technique consists of transplanting tissues dissected from donor embryos into the CAM of recipient embryos. The donor tissue, in contact to the CAM, connects to the recipient vasculature. It allows proper oxygenation of the tissue that can survive and grow autonomously. The use of GFP transgenic chicken allowed an easy tracking of donor cells within the grafted tissue and in the growing embryo recipient (in case of cell migration) (McGrew et al., 2004). Donor hematopoietic tissues (AGM, YS, allantois) were isolated from E3 embryos as the number of IAHC cells peaked in the aorta at this stage (Fig. 2A; Movie 1). Despite established blood circulation between the embryo and the YS at E3, the allantoic rudiment is formed without any vascular connection to the embryo at that stage of development (Caprioli et al., 1998). Transplantations of AGM (Fig. 2B), YS (Fig. 2C) and allantois (Fig. 2D) from E3 GFP^+^ embryos were performed separately into the CAM of E4 wild-type embryo recipients (Fig. 2E-G). Five days post-transplantation, the transplanted tissues were vascularized and were growing (Fig. 2H-J). The transplanted GFP^+^ tissues in the egg can then be readily collected for further analyses (Fig. 2K-M). Of note, the transplanted tissues will thereafter be referred to as tissue CAM (AGM, YS or allantois CAM), whereas the recipients will be referred to as tissue CAM recipients (AGM, YS or allantois CAM recipient).

The percentages of total viable cells (7AAD^−^) and viable hematopoietic cells (7AAD^−^CD45^+^) were evaluated in AGM, YS and allantois at day 0 (post-dissection) and at 5 days (post-transplantation) (Table 1). All tissues were viable [94.0±1.0% (AGM), 69.9±7.6% (YS) and 86.5±7.2% (allantois)] and the percentages of CD45^+^ cells increased in all tissues at 5 days [1.5 and 9% (AGM), 15.1 and 20.5% (YS), 1.6 and 14% (allantois) at day 0 and day 5 post-transplantation, respectively] (Table 1). To determine whether cells from the transplanted tissues could migrate and colonize the recipient, we looked for the presence of GFP^+^ cells in the recipient blood and spleen at 5 days post-transplantation (Fig. 2N). The blood and spleen are the only hematopoietic tissues that can be isolated from E9 embryos (the BM is not formed yet). GFP^+^ cells were present in the blood of AGM and YS CAM recipients but not in allantois CAM recipients (Fig. 2N). Our data thus demonstrate that CAM transplantation is a reliable technique for growing viable embryonic tissues and hematopoietic cells *in ovo*. Moreover, GFP^+^ cells can migrate from the transplanted AGM and YS to the hematopoietic tissues of the recipients.

**Table 1.**
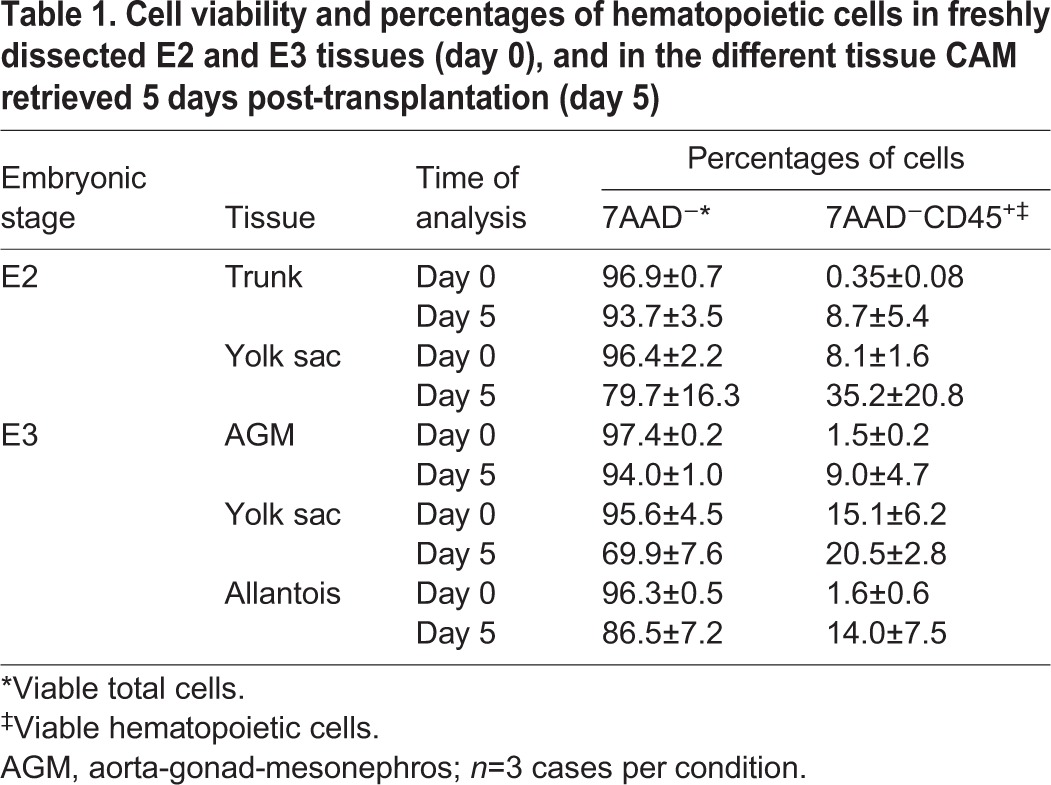
**Cell viability and percentages of hematopoietic cells in freshly dissected E2 and E3 tissues (day 0), and in the different tissue CAM retrieved 5 days post-transplantation (day 5)**

### CAM transplantation reveals the existence of HSCs in the chicken embryo AGM, but not in the yolk sac and allantois

**Fig. 3.**
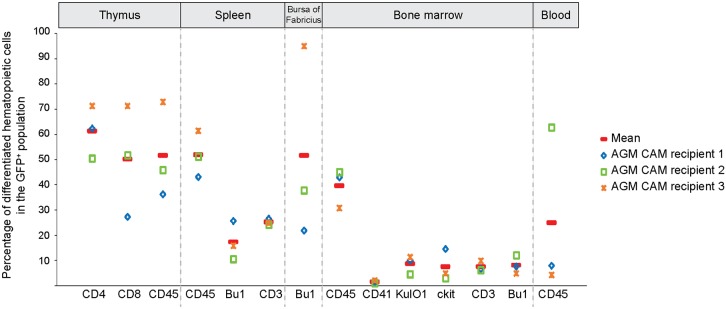
**AGM CAM recipients are repopulated with GFP^+^ cells from lymphoid and myeloid lineages.** Percentages of T cells (CD4^+^, CD8^+^, CD3^+^), B cells (Bu-1^+^), monocytes/macrophages (KUL01^+^), thrombocytes (CD41^+^), hematopoietic stem/progenitor cells (Kit^+^) and total hematopoietic cells (CD45^+^) in the GFP^+^ fraction detected in the thymus, spleen, bursa of Fabricius, BM and blood of three reconstituted AGM CAM recipients. Red line indicates the mean of percentages.

To determine the presence of HSCs in the chicken embryo, the long-term multilineage repopulation capacity of these cells needs to be demonstrated. For this purpose, AGM, YS and allantois were dissected from E3 GFP^+^ embryos and transplanted in the CAM of E4 recipients. The tissue CAM recipients were grown until up to 5 months post-transplantation. To determine the HSC potential of the AGM, YS and allantois, we analyzed the blood, spleen, thymus (T lymphocyte organ), bursa of Fabricius (B lymphocyte organ) and BM of the corresponding CAM recipients for the presence of GFP^+^ cells (Table 2). The reconstituted tissue CAM recipients were further analyzed for multi-lineage repopulation (Fig. 3). The percentage of reconstitution was evaluated both by flow cytometry and PCR in all organs of each tissue CAM recipients. Wild-type adult chickens were used as controls.

**Table 2.**
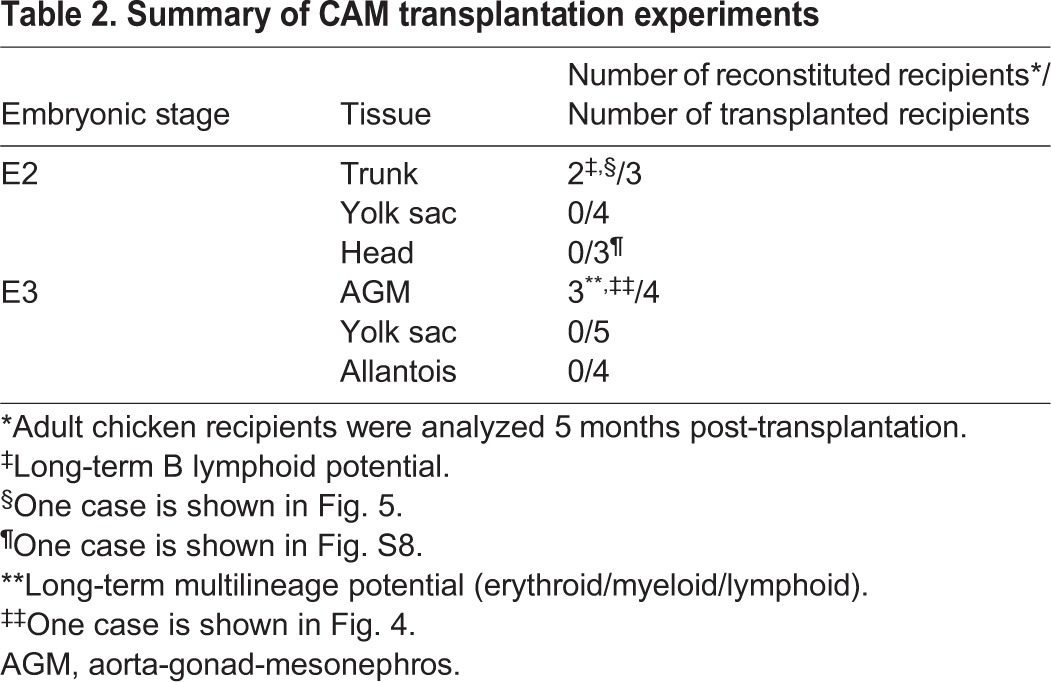
**Summary of CAM transplantation experiments**

**Fig. 4.**
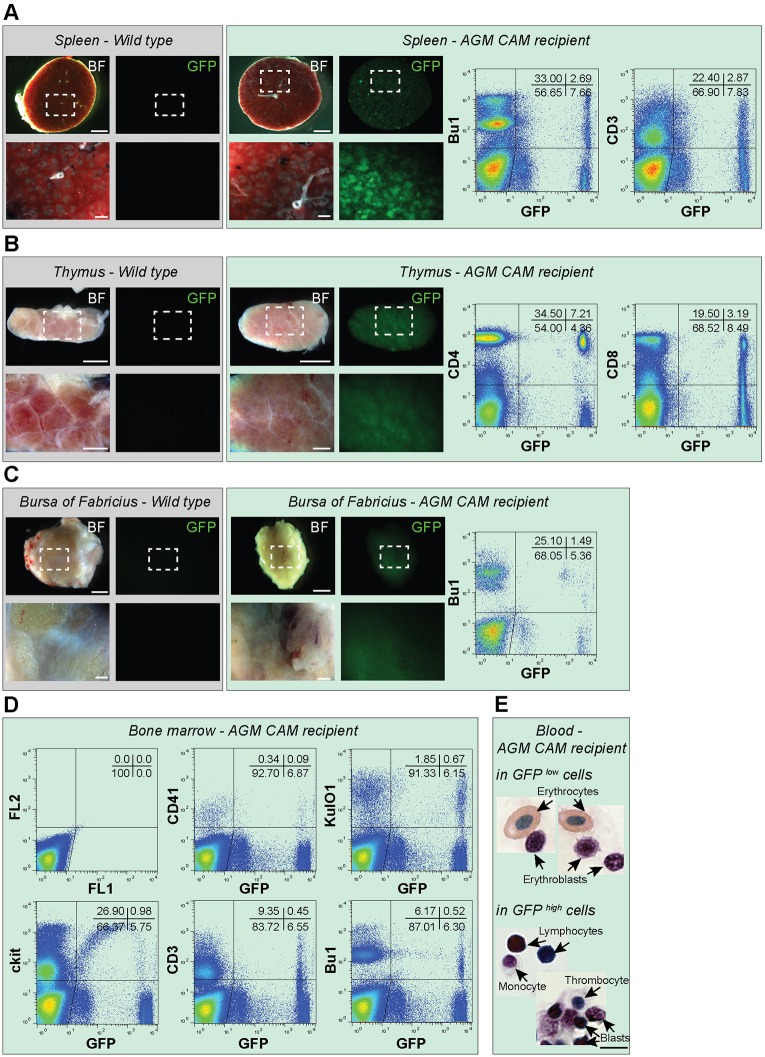
**AGM CAM recipients harbor long-term multilineage reconstitution.** (A-C) Bright filter (BF) and fluorescent (GFP) pictures of spleen (A), thymus (B) and bursa of Fabricius (C) collected from an adult wild-type chicken (gray background) and a reconstituted AGM CAM recipient (green background) at 5 months post-transplantation. Pictures show the whole organ. An enlarged view of the boxed region is shown below each picture. Flow cytometry analysis showing donor-cell contribution (GFP) in the spleen, thymus and bursa of Fabricius of the AGM CAM recipient (right panels in A-C, respectively). Cells were stained using anti-Bu-1 (B cells), anti-CD3 or anti-CD4 and anti-CD8 (T cells) antibodies. (D) Flow cytometry analysis showing donor-cell contribution (GFP^+^) in the BM of the AGM CAM recipient. Cells were stained using anti-CD41 (thrombocytes), anti-KUL01 (monocytes/macrophages), anti-Kit (hematopoietic stem/progenitor cells), anti-CD3 (T cells) and anti-Bu-1 (B cells) antibody. (E) May-Grünwald Giemsa staining of sorted GFP^low^ and GFP^high^ cells collected from the blood of a reconstituted AGM CAM recipient at 5 months post-transplantation. Scale bars: 5 mm in A-C (top pictures); 1 mm in A-C (bottom pictures); 8 µm in E.

Three out of four AGM CAM recipients had GFP^+^ cells in all organs at 5 months post-transplantation (Table 2; AGM CAM recipient 1 is shown as example in Fig. 2O, Fig. 4; AGM CAM recipient 2 is shown as an example in Fig. S2A,B). No GFP^+^ cells were detected in the organs of five YS and four allantois CAM recipients (Table 2; an example is shown in Fig. 2O and Fig. S3). These observations were not a result of defective engraftment as the allantois and YS had grown with a visible functional vascularization at 5 days (Fig. 2I,J,L,M) and 12 days post-transplantation (the allantois CAM is shown as an example in Fig. S4A-D). Our data therefore demonstrate that the AGM is the only organ containing HSCs that is capable of long-term repopulation.

The reconstitution of the three AGM CAM recipients was multilineage with the presence of GFP^+^CD45^+^ hematopoietic cells from the T, B and myeloid lineages in the various hematopoietic organs (Fig. 3). GFP^+^ cells were detected in the spleen, thymic lobes and bursa of Fabricius by microscopic observation (Fig. 4A-C, left panels) and flow cytometry analysis (Fig. 4A-C, right panels). The spleen was colonized by a GFP^+^ population (8.0±4.8%) composed of Bu-1^+^ B cells (17.4±7.7%) and CD3^+^ T cells (25.2±1.2%) (Fig. 3). All thymic lobes contained a GFP^+^ population (8.4±5.0%) composed of CD4^+^ (61.3±10.4%) and CD8^+^ (50.1±22.0%) T cells. The bursa of Fabricius was colonized by a GFP^+^ population (3.9±2.5%) composed of Bu-1^+^ B cells (51.6±38.5%). Finally, the BM harbored a GFP^+^ population (4.6±3.4%) composed of KUL01^+^ monocytes/macrophages (8.6±3.5%), CD3^+^ T cells (7.5±2.0%) and Bu-1^+^ B cells (8.1±3.6%). We also identified CD41^+^ thrombocytes (1.5±0.6%) and Kit^+^ hematopoietic stem/progenitor cells (7.4±6.2%) in the BM (Fig. 3; examples of multilineage analysis are shown in Fig. 4D). Interestingly, GFP^high^ and GFP^low^ donor cells were observed in the organs of the AGM CAM reconstituted recipients. In the blood, GFP^high^ cells were CD45^+^, whereas GFP^low^ cells were CD45^−^ (Fig. 2O). To characterize these two populations, we first examined blood samples of adult wild-type chicken to identify the different cell types. Flow cytometry analysis revealed a very particular arched shape of the blood cells (Fig. S5A). May-Grünwald Giemsa staining allowed us to recognize numerous nucleated erythrocytes (Fig. S5B) and cells resembling to thrombocytes, lymphocytes and myeloid (eosinophil) cells (Fig. S5C). CD4/CD8^+^ T cells, Bu-1^+^ B cells, CD41^+^ thrombocytes and KUL01^+^ monocytes/macrophages were then sorted and stained subsequently to combine cell identity (immunophenotype) and cell morphology (Fig. S5D-G, respectively). GFP^low^ and GFP^high^ cells were then sorted from the blood of AGM CAM recipient 1. Observation of the sorted cells after May-Grünwald Giemsa staining demonstrated that GFP^low^ cells were erythrocytes and erythroblasts, whereas GFP^high^ cells were non-erythroid cells such as lymphocytes, monocytes/macrophages, thrombocytes and immature hematopoietic (blast) cells (Fig. 4E).

Our data demonstrate that the AGM, which contains IAHC and PAF cells, is able to provide long-term multilineage hematopoietic reconstitution upon CAM transplantation. This was not the case for the YS and allantois. Therefore, HSCs are present only in the AGM of E3 chicken embryos.

### Long-term B-lymphoid potential in E2 embryos

**Fig. 5.**
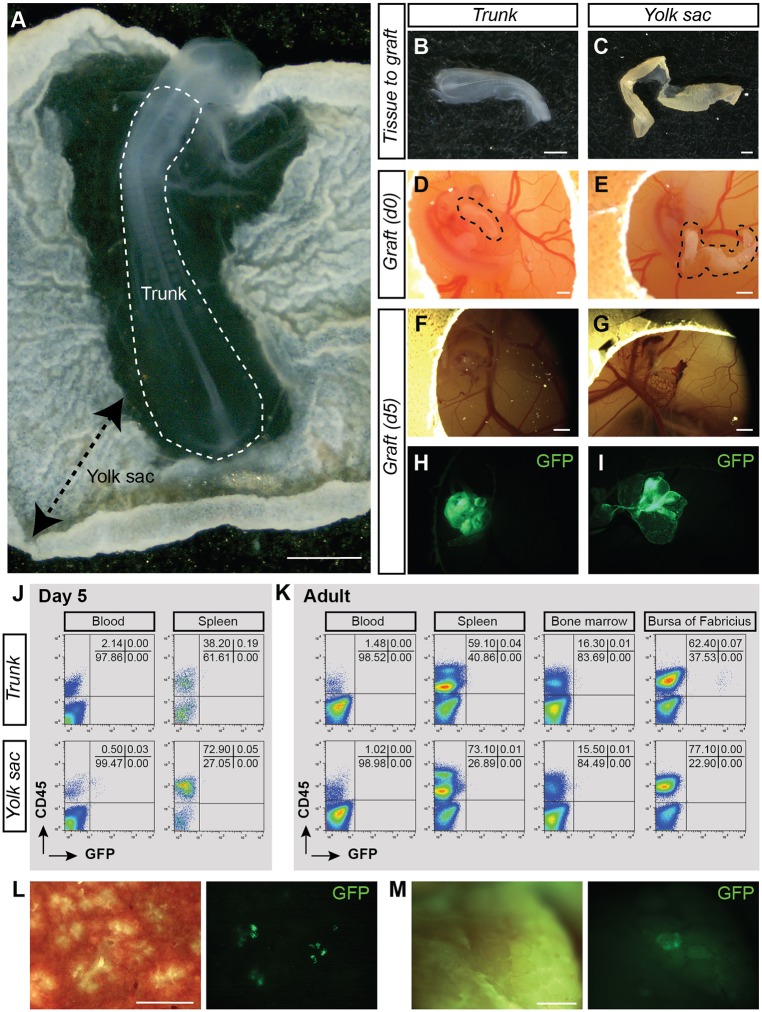
**Long-term B-lymphoid biased potential in E2 embryo.** (A) Transmitted light picture of an E2 GFP^+^ chicken embryo. Trunk and yolk sac (YS) locations are indicated. (B,C) Transmitted light pictures of the trunk (B) and YS (C) after dissection. (D,E) Transmitted light pictures of the trunk (D) and YS (E) (shown in B and C) after transplantation in the CAM of E4 recipient embryos. Dashed areas indicate grafted tissues. (F,G) Transmitted light pictures of the trunk (F) and YS (G) CAM at 5 days post-transplantation. (H,I) Fluorescent (GFP) pictures of the trunk (H) and YS (I) CAM shown in F,G. (J) Flow cytometry analysis showing donor-cell contribution (GFP) in blood and spleen of trunk (top plots) and YS (bottom plots) CAM recipients at 5 days post-transplantation. Cells were stained with anti-CD45 (hematopoietic cells) antibody. (K) Flow cytometry analysis showing donor-cell contribution (GFP) in blood, spleen, bone marrow and bursa of Fabricius of trunk (top plots) and YS (bottom plots) CAM recipients at 5 months post-transplantation. Cells were stained with anti-CD45 (hematopoietic cells) antibody. The analyzed tissue CAM recipient is shown in Table 2. (L) Enlarged view of a transverse section of the spleen isolated from an adult trunk CAM recipient [transmitted light, left panel; fluorescence (GFP), right panel]. (M) Enlarged view of the bursa of Fabricius isolated from an adult trunk CAM recipient [transmitted light, left panel; fluorescence (GFP), right panel]. Scale bars: 1 mm.

To determine whether cells with a HSC potential could be detected at an earlier time point of development, CAM transplantations were performed with E2 GFP^+^ YS and embryo trunk (Fig. 5A). At E2, no IAHC and PAF cells are detected in the aorta, and the allantois is not formed yet (Movie 2). The trunk (embryo without heart and head) and the YS were dissected from GFP^+^ embryos (Fig. 5B,C), and transplanted into the CAM of E4 wild-type recipients (Fig. 5D,E; Table 2). Five days post-transplantation, trunk and YS were incorporated in the CAM, had vascularized and had grown (Fig. 4F-I). The GFP^+^ trunk and YS CAM were viable [Table 1; 93.7±3.5% (trunk) and 79.7±16.3% (YS)] and the percentages of CD45^+^ cells had increased [Table 1; from 0.35 to 8.7% (trunk) and from 8.1 to 35.2% (YS)]. Therefore, the CAM transplantations allow the development of E2 trunk and YS *in ovo*. The blood and spleen of the tissue CAM recipients were analyzed by flow cytometry at 5 days post-transplantation (Fig. 5J). No GFP^high^ cells were detected in three YS and three trunk CAM recipients (Table 2).

To decipher whether E2 tissues are too immature to produce a hematopoietic progeny in CAM recipients (compared with E3 tissues), three trunk and four YS CAM recipients were analyzed at 5 months post-transplantation by flow cytometry and PCR (Table 2; Fig. S6). No GFP contribution was observed by PCR in the hematopoietic organs of YS and trunk CAM recipients (an example of each type of recipient is shown in Fig. S6). Interestingly, few CD45^+^GFP^high^ cells were detected in the spleen and bursa of Fabricius in two out of three trunk CAM recipients after flow cytometry analysis and microscopic observation of the organs (an example is shown in Fig. 5K-M). Of note, GFP in these organs was below the detection limit by PCR (Fig. S6). As expected, the GFP^high^ cells in the spleen and bursa of Fabricius were Bu-1^+^ B cells (Fig. S7).

As the presence of HSCs was detected in the head as early as in the AGM in the mouse embryo (E10.5) (Li et al., 2012), we also tested the HSC potential of the chicken embryonic head. GFP^+^ E2 chicken heads were transplanted in the CAM of E4 wild-type recipients (Fig. S8A). Despite a successful engraftment of the head, as shown by the growing of the head in the CAM (Fig. S8A-D), GFP^+^ cells were neither found circulating in the blood nor in the hematopoietic organs of three head CAM recipients 5 months post-transplantation (Fig. S8E; Table 2).

Altogether, our data show that the YS and the head do not contain cells with long-term multi-lineage hematopoietic reconstitution at E2. Interestingly, the embryo proper is either able to produce biased B-lymphoid stem cells or long-lived B cells (cells able to survive in a competitive environment for up to 5 months post-transplantation).

## DISCUSSION

Avian and human embryos share particular features that are not always found in other species. Whereas IAHCs are restricted to the aortic floor in human and chicken embryos (Jaffredo et al., 1998; Tavian et al., 1996), they are also present in the aortic roof in mouse (Taoudi and Medvinsky, 2007). In mouse embryos, IAHCs emerge as individual clusters of up to 19 cells, implying a dispersion of HE cells within the aortic endothelium (Boisset et al., 2015; Yokomizo and Dzierzak, 2010). In contrast, a large proportion of the aortic floor becomes hematopoietic in avian and human embryos, in which EHT causes thickening of the endothelium rather than the production of individual IAHCs (Bollerot et al., 2005; Tavian et al., 1996). The similarities between human and chicken embryos, and the difficulties to use early human embryos suggest the chicken embryo as a reliable attractive alternative, or even a model of choice. However, the hematopoietic development of the avian embryo has not been studied as precisely as in other species. We therefore established the complete cartography of the hematopoietic cell production in the chicken embryo aorta during development. We also demonstrated the existence of long-term multilineage HSCs originating from the AGM (and not from the YS, allantois or head), by performing the first *in ovo* transplantation of donor GFP^+^ embryonic tissues on the CAM of recipient embryos that were analyzed in adulthood.

The discovery of IAHCs in the main arteries of most mammalian embryos was of historical importance as HSCs and pre-HSCs locate in IAHCs (Boisset et al., 2015; Rhodes et al., 2008; Taoudi et al., 2008; Tavian et al., 1996; Yokomizo and Dzierzak, 2010). Similar to humans, IAHC cells in chicken emerge exclusively in the floor of the aorta, as previously described on histological embryo sections (Jaffredo et al., 2000, 1998; Tavian et al., 1996). We show here that IAHC cells indeed emerge from the floor of the aorta but only in the anterior region, restricted by the aortic arches and the aorta-vitelline connection. IAHC emergence occurs as a wave along the anterior to posterior axis following or as a consequence of the two bilateral aortic anlagen fusion, also occurring along the anterior to posterior axis as the embryonic development unfolds. The wave stops at the level of the aorta-vitelline connection. Indeed, no IAHCs were detected below the connection or in the vitelline arteries, whereas they are numerous in E9 mouse embryo vitelline and umbilical arteries (Yokomizo and Dzierzak, 2010). Interestingly, potent HE cells (Runx1^+^MEP21^+^) were present posterior to and in the region of the aorta-vitelline connection, as well as in E2 chicken aorta. It therefore indicates a strong spatial and temporal regulation of HE cells by the surrounding microenvironment. Such regulation is less obvious in the mouse embryo where IAHCs are present in both sides of the aorta (although less numerous in the roof) (Taoudi and Medvinsky, 2007). Moreover, the HSC potential is not restricted to ventral IAHCs (Souilhol et al., 2016). Because the spatial and temporal IAHC cartography is now precisely established in the chicken embryo, it could be used in the future to study the role of the surrounding microenvironment in promoting or preventing IAHC (and HSC) emergence in specific areas of the aorta.

The correlation between the cells emerging in the aorta and the number of true HSCs has been recently made in the zebrafish model (Henninger et al., 2016). Using a Zebrabow system, 30 clones (and therefore 30 HSCs) were shown to establish the entire hematopoietic system. Because an average of 60 cells emerge in the aorta, it suggests that half of them would be potent HSCs. In the mouse, 700 IAHC cells are present at E10.5 (Yokomizo and Dzierzak, 2010). In comparison, human (day 35) and chicken (E3) embryos have thousands of IAHC cells (Tavian et al., 1999). However, the estimated number of HSCs is less than three per mouse or human AGM (Ivanovs et al., 2011; Kumaravelu et al., 2002; Tavian et al., 2001). It might indicate that either the HSC numbers in mammals are underestimated (owing to the transplantation assay) or that the numbers of IAHC cells and HSCs are not correlated in mammals. The improvement of techniques to isolate, to trace and/or to assay HSCs will certainly help to discriminate between these two hypotheses in the future.

Following emergence, IAHC cells are known to ingress in the mesenchyme underneath the aorta to form PAFs (Dieterlen-Lièvre and Martin, 1981; Jaffredo et al., 2000). However, a precise mapping of these cells during embryo development was missing. Cells located in PAF were initially described as lymphoid stem cells (Lassila et al., 1980) that were responsible for the first colonization wave of the thymus (Dunon et al., 1998, 1999; Lassila et al., 1980). Cells would expand in PAFs (considered as the mammalian fetal liver equivalent) before colonizing adult hematopoietic tissues such as BM, thymus, bursa of Fabricius and spleen, most likely through the bloodstream, as demonstrated by quail-chicken transplantation experiments (Le Douarin et al., 1975, 1976; Le Douarin and Jotereau, 1975; Dieterlen-Lievre, 1975). PAF cells also emerge as a wave along the anterior to posterior axis of the embryo. However, they do not completely follow the same distribution as IAHC cells as PAF cells were more homogeneously distributed along the aorta. It might reflect the temporary accumulation of PAF cells underneath the aorta, while IAHC cell emergence stops there and starts in a more posterior region of the aorta as embryonic development progresses. The number of PAF cells largely surpasses the number of IAHC cells, with up to 6000 PAF cells in E4 aorta. However, fewer than 5% of PAF cells proliferated. PAFs are therefore not a site where cells actively proliferate but rather a site where IAHC cells progressively accumulate and mature. In addition, the possibility cannot be totally excluded that HE cells might produce cells that immediately ingress in the sub-aortic mesenchyme without emerging first towards the aortic lumen. In mouse embryo, IAHC cells colonize the fetal liver via the bloodstream where they mature to participate to the formation of the HSC pool (Rybtsov et al., 2016). Similarly, PAFs might be a transient site where cells mature before colonizing the thymus (Lassila et al., 1980) and the bursa of Fabricius.

No assay demonstrating both long-term and multipotent potential of embryonic cells/tissues was available to test the existence of bona fide HSCs in birds. Here, we demonstrate for the first time the existence of HSCs capable of long-term (up to 5 months post-transplantation) and multilineage (erythroid, lymphoid and myeloid) reconstitution after the transplantation of GFP^+^ AGM isolated at E3 (when IAHCs/PAFs are present) in the CAM of wild-type embryos. Despite efficient engraftments, no reconstitution was observed in the recipients transplanted with YS or the allantoic rudiment, therefore demonstrating the absence of HSCs in these tissues at E3. HSC detection in the embryo seems concomitant with the presence of IAHC and PAF cells in the aorta as HSCs were not detected when GFP^+^ trunks isolated at E2 (no IAHCs/PAFs yet) were transplanted. The absence of markers to discriminate IAHC and PAF cells does not yet permit to determine whether HSCs are already present in IAHCs or whether they need to ingress in PAFs to acquire this potential. Besides, the transplantation of IAHC or PAF cells would be extremely challenging. Before performing CAM transplantation, we tried to inject sorted GFP^+^CD45^+^ IAHC/PAF cells or total cells (isolated from GFP^+^ allantois or YS) directly in the bloodstream or in the ventral mesenchyme underneath the aorta of E3 recipients. Despite a very low donor cell contribution (up to 0.6% of GFP^+^ cells) in the recipients analyzed 12 days post-transplantation (i.e. E16), no donor cells were ever detected in the transplanted recipients at the adult stage (0/8 recipients analyzed 5 months post-transplantation).

Interestingly, a long-term B lymphoid reconstitution was observed in some CAM recipients transplanted with GFP^+^ trunks isolated at E2, revealing the presence of intra-embryonic biased B-lymphoid stem cells or long-lived B lymphoid precursors at this early stage of development. It is in accordance with the previous identification of B lymphoid precursors in the aortic region of the mouse embryo, as shown after *in vitro* culture of E8.5-E9.5 cells on BM stromal cell lines (Cumano et al., 1993; Ogawa et al., 1988), or after *in vivo* transplantation in immunodeficient adult (Godin et al., 1993) or neonatal mice (Yoshimoto et al., 2011). Interestingly, E9-E9.5 YS have also the autonomous potential to produce B progenitors, providing B cell long-lived progeny upon transplantation directly into the peritoneal cavity of neonates (Yoshimoto et al., 2011). Our data indicate that such potential is not present (or is too low to be detected) in the chicken YS. In addition, no HSC potential was detected in the head of the chicken embryo, although it has been reported previously in the mouse embryonic head (Li et al., 2012), indicating that such potential might be species restricted. Overall, our CAM transplantation allowed: (1) the transplantation and survival of embryonic tissues; (2) the migration and colonization of donor cells in the embryo recipient; and (3) the ability of potent grafted cells to provide a long-term hematopoietic production.

Our study extends the knowledge about the hematopoietic (stem) cell production as it occurs in the aorta of the chicken embryo *in vivo*. The precise organization of hematopoietic cells along the aorta being now well documented, the chicken model can further be used to study the regulation of IAHC/HSC in the embryo. Combining the knowledge on chicken hematopoiesis, the HSC transplantation assay and gain/loss-of-function experiments (e.g. to target potential regulatory genes via *in ovo* electroporation of the aorta, sh-RNA inoculation or CRISPR-cas9 technology) should provide new insights into the regulation of HSCs *in vivo*. Accordingly, the avian model represents an exciting and powerful model to open new paths for future exciting breakthrough discoveries.

## MATERIALS AND METHODS

### Chicken embryo generation

Fertilized wild-type chicken eggs (Bovans Brown; Het Anker poultry, The Netherlands) were incubated at 37±1°C in a humidified incubator until reaching the appropriate developmental stage [embryonic day (E)2-E5.5]. E4 wild-type embryos served as recipients for the chorio-allantoic membrane (CAM) transplantations. E2 and E3 GFP^+^ transgenic eggs (Roslin Institute, University of Edinburgh, UK) were used as donors. After hatching, tissue CAM recipients were housed at the Gemeenschappelijk Dierenlaboratorium facility (Utrecht, The Netherlands) until they reached adulthood. Animals were housed according to institutional guidelines, and procedures were performed in compliance with Standards for Care and Use of Laboratory Animals, with approval from the Dutch Animal Experiment Committee.

### CAM transplantations

E2 and E3 GFP^+^ donor eggs were used. The embryo was detached from the yolk and washed with PBS (Figs 2A and 5A). Embryo and YS were then isolated using forceps and scissors (Figs 2B,C and 5B,C). In some cases, heads were also isolated from E2 GFP^+^ embryos. Because the allantoic rudiment forms later on during development, it was only isolated at E3, before the blood vascularization was established with the embryo (Fig. 2D) (Caprioli et al., 1998). Intact GFP^+^ tissues were grafted into the CAM of E4 wild-type embryos that were used as recipients (Figs 2E-G and 5D,E; Fig. S8A-D). An incision was made in the CAM recipient with an insulin needle before gently inserting the tissues into the CAM incision. Then, the window in the egg shell was sealed with tape and the egg was placed back in the incubator for further development of the embryo.

### Tissue CAM viability and short-term engraftment analysis of tissue CAM recipients (at 5 days post-transplantation)

Tissue CAM were analyzed at 0 day (on freshly dissected tissues) and 5 days post-transplantation. Notably, the allantois CAM was analyzed at 9 days. Trunks, AGMs, YSs and allantoic rudiments were collected, and cell suspensions were obtained by collagenase treatment [45 min at 37°C, collagenase Type I, (Sigma, C-0130)]. After washing with PBS (supplemented with 10% of fetal calf serum, FCS; PBS/FCS) and filtering, cells were stained with PE anti-CD45 antibody. 7-Aminoactinomycin D (7-AAD) was used to determine cell viability. Donor cell migration and colonization in the tissue CAM recipients was evaluated by measuring the percentage of GFP (by flow cytometry) in the peripheral blood and spleen of the recipients.

### Long-term engraftment analysis of tissue CAM recipients (at 5 months post-transplantation)

Egg incubation was prolonged until hatching and growing chicken were analyzed at 5 months post-transplantation to test long-term reconstitution potential. The peripheral blood was collected from the brachial vein into 1.3 ml collection tube containing EDTA. Cells were washed in PBS/FCS and stained with antibodies for 20 min at 4°C (antibodies are listed in Table S1). Multilineage reconstitution was analyzed in hematopoietic organs (thymus, spleen, bursa of Fabricius and BM). Organs were collected, crushed and cell suspensions were filtered (40 µm nylon cell strainer, Falcon) and washed twice in PBS/FCS. Single-cell suspensions were then stained (Table S1). 7-AAD was used to exclude dead cells. Flow cytometry analyses were performed on a FACSCalibur. Data were analyzed using FlowJo software.

### Immunostaining on whole-mount chicken embryo and confocal imaging

#### Embryo preparation

Embryos were prepared as previously described (Boisset et al., 2011; Yokomizo and Dzierzak, 2010), with few modifications. Briefly, embryos were gently perfused in the heart with purified anti-MEP21 antibody (McNagny et al., 1992) to flush out the blood (via the vitelline vessels) and to stain the entire vascular endothelium. Embryos were then washed three times in PBS before fixation in PBS supplemented with 4% PFA (PBS/PFA) for 20 min at 4°C. Embryos were washed in PBS before a dehydration-rehydration step in methanol. Embryos were incubated in PBS supplemented with 1% skim milk, 0.4% TritonX-100, 0.2% BSA and 0.1% goat serum (PBS-MT) for 1 h at 4°C. Each antibody [primary and secondary (Table S2)] incubation was performed overnight at 4°C in PBS-MT, followed by three washes in PBS-MT the following day. At the end of the staining procedure, embryos were washed three times in PBS supplemented with 0.4% of TritonX-100 three times for 20 min. Embryos were then dehydrated in methanol and cleared progressively via successive passages into methanol/50% BABB (benzyl alcohol:benzylbenzoate, 1:2) and 100% BABB solutions. Transparent embryos were mounted between slide and coverslip by using Fast Wells (Grace Bio-Labs).

#### Embryo/organ imaging and analysis

Transparent embryos and aortas were imaged with a Zeiss LSM700 confocal microscope (10×PlanApo dry objective). The pictures of whole organs and whole chicken embryos in eggs were taken using a Leica stereomicroscope MZ16FA with a Hamamatsu Flash4 LT digital camera. Three-dimensional reconstructions were generated from z-stacks with Imaris software and converted to QuickTime files. IAHC and PAF cells were counted by using the Zeiss Zen (blue edition) software. Proliferating (PHH3^+^) IAHC and PAF cells were counted to determine the mitotic index (MI=number of PHH3^+^ IAHC or PAF cells/total number of IAHC or PAF cells×100).
